# PFAS Concentrations and Cardiometabolic Traits in Highly Exposed Children and Adolescents

**DOI:** 10.3390/ijerph182412881

**Published:** 2021-12-07

**Authors:** Cristina Canova, Andrea Di Nisio, Giulia Barbieri, Francesca Russo, Tony Fletcher, Erich Batzella, Teresa Dalla Zuanna, Gisella Pitter

**Affiliations:** 1 Unit of Biostatistics, Epidemiology and Public Health, Department of Cardio-Thoraco-Vascular Sciences and Public Health, University of Padova, 35131 Padova, Italy; giulia.barbieri.1@unipd.it (G.B.); erich.batzella@unipd.it (E.B.); teresa.dallazuanna@studenti.unipd.it (T.D.Z.); 2 Unit of Andrology and Reproductive Medicine, Department of Medicine, University of Padova, 35131 Padova, Italy; andrea.dinisio@unipd.it; 3 Eurac Research, Institute for Biomedicine, 39100 Bolzano, Italy; 4 Directorate of Prevention, Food Safety, and Veterinary Public Health-Veneto Region, 30123 Venice, Italy; francesca.russo@regione.veneto.it; 5 Public Health, Environments and Society Department, London School of Hygiene and Tropical Medicine, London WC1H 9SH, UK; tony.fletcher@lshtm.ac.uk; 6 Screening and Health Impact Assessment Unit, Azienda Zero-Veneto Region, 35131 Padua, Italy; gisella.pitter@azero.veneto.it

**Keywords:** perfluoroalkyl substances (PFAS), children, adolescents, lipid profile, cholesterol, generalized additive model (GAM), weighted quantile sum (WQS) regression, mixture

## Abstract

Background: Residents of a large area of north-eastern Italy were exposed for decades to high concentrations of perfluoroalkyl and polyfluoroalkyl substances (PFAS) via drinking water. Despite the large amount of evidence in adults of a positive association between serum PFAS and metabolic outcomes, studies focusing on children and adolescents are limited. We evaluated the associations between serum PFAS concentrations that were quantifiable in at least 40% of samples and lipid profile, blood pressure (BP) and body mass index (BMI) in highly exposed adolescents and children. Methods: A cross-sectional analysis was conducted in 6669 adolescents (14–19 years) and 2693 children (8–11 years) enrolled in the health surveillance program of the Veneto Region. Non-fasting blood samples were obtained and analyzed for perfluorooctanoic acid (PFOA), perfluorooctane sulfonate (PFOS), perfluorohexanesulfonic acid (PFHxS), perfluorononanoic acid (PFNA), total cholesterol (TC), high-density lipoprotein cholesterol (HDL-C) and triglycerides. Low-density lipoprotein cholesterol (LDL-C) was calculated. Systolic and diastolic BP were measured, and BMI z-score accounting for age and sex was estimated. The associations between ln-transformed PFAS (and categorized into quartiles) and continuous outcomes were assessed using generalized additive models. The weighted quantile sum regression approach was used to assess PFAS-mixture effects for each outcome. Analyses were stratified by gender and adjusted for potential confounders. Results: Among adolescents, significant associations were detected between all investigated PFAS and TC, LDL-C, and to a lesser extent HDL-C. Among children, PFOS and PFNA had significant associations with TC, LDL-C and HDL-C, while PFOA and PFHxS had significant associations with HDL-C only. Higher serum concentrations of PFAS, particularly PFOS, were associated with lower BMI z-score. No statistically significant associations were observed between PFAS concentrations and BP. These results were confirmed by the multi-pollutant analysis. Conclusions: Our study supports a consistent association between PFAS concentration and serum lipids, stronger for PFOS and PFNA and with a greater magnitude among children compared to adolescents, and a negative association of PFAS with BMI.

## 1. Introduction

Over the past few decades, per- and polyfluoroalkyl substance (PFAS) contamination has grown into a serious global health threat. PFAS are used in numerous consumer products and industrial applications to confer waterproof, greaseproof, stain-proof and low-friction properties [1]. PFAS are among the most ubiquitous synthetic chemicals in the world, and environmental and human exposure to PFAS can occur throughout the life cycles of these chemicals and products containing them [2]. Humans are exposed to PFAS via ingestion of contaminated food and drinking water [3], inhalation of indoor air and indoor dust [4] and use of consumer products. In humans, PFAS half-lives in serum may vary greatly with expected variation in children (small blood volumes and large fraction of exposures coming from drinking) compared to adults [5]. Among the numerous PFAS congeners, only a few PFAS have been thoroughly studied from an epidemiological perspective, especially perfluorooctanoic acid (PFOA) and perfluorooctane sulfonate (PFOS). Adverse impacts have been consistently reported for PFOA and PFOS on the endocrine, immune, and metabolic outcomes (lipid profile, blood pressure, obesity and metabolic syndrome) in occupationally exposed, highly exposed, and general populations [6]. Despite the large amount of evidence in adults, studies focusing on the association between serum PFAS and metabolic outcomes in children and adolescents are limited, and the potential associations for some outcomes have been scarcely studied in European child and adolescent populations [7,8,9].

Given the emerging consensus that the development of cardiovascular disease in adulthood is preceded by metabolic changes occurring in childhood, and considering interspecies and interindividual differences in concentration–response assessment and different exposure conditions, it is important to identify the risk associated with exposure to PFAS in this population. Because both childhood and adolescence are distinguished by marked changes in growth, sexual maturity and hormonal secretion, the risk of endocrine disruption by extrinsic agents may differ during these life phases [10]. Furthermore, the number of underlying factors (e.g., prevalent chronic or acute disease or medication use) confounding the associations between environmental exposure and potential health consequences in children and adolescents is likely to be smaller in this population and is worthy of further insight. 

Between the late 1960s and 2013, residents of 30 municipalities across the provinces of Vicenza, Padova and Verona in the Veneto Region (north-eastern Italy), labelled as Red Area, were exposed to high concentrations of PFAS, particularly PFOA, via contaminated drinking water from a manufacturing plant [3]. In this context, the objective of this study was to evaluate the associations between serum PFAS concentrations and various cardiometabolic traits (lipid profile, blood pressure, and body mass index) in a large group of highly exposed adolescents aged 14–19 years and children aged 8–11 years recruited as part of a community-based health surveillance program in Veneto Region, Italy [3].

## 2. Materials and Methods

### 2.1. Study Design and Recruitment

The health surveillance program in Veneto Region is an ongoing cross-sectional study that started in 2017; it is used to document exposure to PFAS and to evaluate associated adverse health outcomes in a highly exposed group of community residents of the Reda Area. The project has been more completely described elsewhere [3]. The participants in this study were 9475 adolescents aged 14 to 19 years and children aged 8 to 11 years at their enrollment in the health surveillance program, which happened between January 2017 and December 2019. It represents one of the largest community-based studies to date investigating potential associations between PFAS exposure and human health effects in these age groups. Potential participants were sent an invitation letter that explained the health surveillance program and fixed an appointment. Participants <19 years old had to be accompanied by a parent or legal guardian. To facilitate recruitment at different times of the day while minimizing the research impact on school attendance, participants were not required to be in fasting conditions. Informed consent from the participants or their parents/legal guardians was obtained orally and recorded in the individual’s clinical charts.

### 2.2. Data Collection

At four different centers (Lonigo, Legnago, San Bonifacio, and Noventa Vicentina), trained nurses collected personal and medical data, anthropometric measures (height, weight), blood pressure and blood samples from the participants. Analyses of clinical biomarkers, including serum lipids, were carried out from three different laboratories (Arzignano, San Bonifacio, Legnago). Medical history, medications, self-reported height and weight, socio-demographic information, and lifestyle habits were collected using interviewer-administered questionnaires. 

### 2.3. Outcomes of Interest

#### 2.3.1. Lipid Profile

Several plasma lipid parameters, including total cholesterol (TC), high-density lipoprotein cholesterol (HDL-C), and low-density lipoprotein cholesterol (LDL-C), were measured by a direct enzymatic colorimetric assay using cholesterol esterase and cholesterol oxidase. Triglycerides were measured using an assay based on glycerolphosphate oxidase-peroxidase aminophenazone. The measurement of serum lipids was performed in a Cobas automated clinical chemistry analyzer (Roche Diagnostics GmbH, Mannheim, Germany) in two laboratories and in an AU automated clinical chemistry analyzer (Beckman-Coulter, Brea, CA, USA) in the third laboratory. The three laboratories regularly follow an external quality assurance program. LDL-C was calculated by the Friedewald equation when triglycerides were less than 400 mg/dL (for 16 subjects it was not possible to calculate it). 

#### 2.3.2. Overweight and Obesity

BMI was recalculated after checking the accuracy of data regarding the self-reported height and weight. Since the relationship between weight and height changes considerably during the childhood, we converted BMI to a BMI z-score accounting for age and sex using the recommended WHO Growth References for school-aged children and adolescents [11]. The Z-score system expresses the anthropometric value as a number of standard deviations or Z-scores below or above the reference mean or median value [12]. A BMI z-score of ≥2 indicates obesity.

This measure was obtained using the R package z-scorer, calculated as a number of standard deviations above or below the reference median value, based on the WHO Growth References for school-aged children and adolescents (https://www.who.int/nutgrowthdb/about/introduction/en/index4.html, accessd on 15 November 2021).

#### 2.3.3. Blood Pressure 

Blood pressure (BP) was measured by trained nurses with participants first sitting at rest for at least five minutes, according to the European Society of Hypertension recommendations. A validated semi-automatic sphygmomanometer with an appropriate cuff size for the arm circumference was used. If the first measure was above normal values for age, gender and height, a second measurement was taken at least two minutes apart. 

### 2.4. Serum PFAS Measurement

Serum concentrations of twelve PFAS, consisting of PFOS, PFOA, perfluorohexanesulfonic acid (PFHxS), perfluorononanoic acid (PFNA), perfluoroheptanoic acid (PFHpA), perfluorobutanesulfonic acid (PFBS), perfluorohexanoic acid (PFHxA), perfluorobutanoic acid (PFBA), perfluoropentanoic acid (PFPeA), perfluorodecanoic acid (PFDeA), perfluoroundecanoic acid (PFUnA), and perfluorododecanoic acid (PFDoA), were measured by high-performance liquid chromatography coupled with triple quadrupole mass spectrometry (HPLC MS/MS, Shimadzu UFLC XR 20 Prominence coupled to Sciex API 4000). Details of the analytical procedure for measuring the twelve PFAS in serum samples have been described previously [3]. Method performances allow analytes to be detected as low as 0.1 ng/mL (LOD) and to be quantified above 0.5 ng/mL (LOQ). Following published practices, levels less than the LOQ were imputed to be LOQ/√2, and we limited our statistical analyses to PFAS detected in ≥40% of the overall samples. Three of the twelve PFAS were detected in more than 98% of the serum specimen: PFOA (detected in 99.9% of adolescents and children), PFOS (detected in 99.8% of adolescents and 99.3% of children), and PFHxS (detected in 97.6% of adolescents and 96.6% of children). PFNA was detected in 43.8% of adolescents and 28.3% of children. Detection rates of all PFAS are presented as supplementary material, Table S1.

### 2.5. Covariates

Potential confounders were selected through the construction of a directed acyclic graph (DAG) based on existing literature, and the identification of a minimally sufficient set of variables to control confounding. The minimally sufficient adjustment set was identified using DAGitty v1.0 (www.dagitty.net, accessed on 15 November 2021) implemented in R (R Development Core Team 2010, R Foundation for Statistical Computing, Vienna, Austria. ISBN 3-900051-07-0, URL: http://www.R-project.org/, accessed on 15 November 2021). The DAG is presented in supplementary material, Figure S1.

The final model for adjustment included age, gender, country of birth, data on food consumption, degree of physical activity, salt intake, smoking status (for adolescents only), time-lag between the beginning of the study and the date of enrollment. Frequency of food consumption was collected for the following food categories: meat, fish/seafood, milk/yogurt, cheese, eggs, bread/pasta/cereals, sweets/snacks/sweet beverages, fruits/vegetables, and salt. Data on food consumption were transformed from number of servings per day/week/month to number of servings per week for all the food categories to create harmonized diet pattern classification. Smoking status was subdivided into current smokers, previous smokers and non-smokers. Degree of physical activity (Light, Moderate, or Heavy) was defined based on an algorithm that combined information reported by the subject on intensity, duration, and frequency of all types of physical activity practiced during the week. Countries of birth were classified in two categories based on geographical areas, including: Italy plus other Highly Developed Countries, and High Migratory Pressure Countries. The time-lag between the beginning of the study (1 January 2017) and the date of enrollment was calculated for each subject and included as a possible covariate (number of months).

Information on the center in charge of the blood pressure and anthropometric measurements was considered as a possible confounder in statistical analyses on blood pressure and BMI. Lipid models, instead, were adjusted by a laboratory in charge of the analyses of clinical biomarkers. Analyses on lipids and blood pressure were also adjusted for the BMI values.

### 2.6. Statistical Analysis

After excluding pregnant teenagers and subjects with incomplete exposure or outcome data (supplementary material, Figure S2), 6669 adolescents and 2693 children were included in the statistical analyses. The serum concentrations of PFAS were expressed as arithmetic mean, standard deviation (SD) and percentiles. Spearman’s correlation (ρ) was used to describe pair-wise relations between the PFAS. 

All continuous outcomes (Total Cholesterol, HDL Cholesterol, LDL Cholesterol, Systolic and Diastolic Blood Pressure), except BMI z-score, were analyzed considering a different subset of observations, excluding those with specific self-reported diseases and medications. Models for lipid outcomes were fitted excluding subjects under statin drugs or other cholesterol-lowering medications, and models for blood pressure outcomes were fitted excluding subjects under anti-hypertensive drugs or with self-reported hypertension. PFOA, PFOS, PFHxS and PFNA were treated as both continuous, to determine the association between the outcome and the PFAS concentration by linear regression analysis, and categorical-quartiles of exposure, with the lowest PFAS quartile as a reference group in order to examine any dose–response trends and limit the influence of extreme values. For PFNA, due to the high proportion of the measures below the LOQ, PFNA was classified in three categories: below the limit of quantification, low and medium. The latter two were defined using a cut-off based on the median of the distribution of PFNA above the limit of classification. For analysis as a continuous variable, PFAS values were natural log (ln)-transformed to correct skewed distribution and improve normality of the data. 

The relation between each ln-transformed PFAS and continuous outcomes was analyzed using Generalized Additive Models (GAMs). Thin plate spline smooth terms were used for the exposure and continuous covariates, in order to explore non-linear shapes of possible association between PFAS and outcomes. Degree of smoothing was selected by generalized cross-validation, as implemented in the R package mgcv. The interpretation of spline analyses does not produce interpretable coefficients besides of EDF, which represents the degree that a polynomial function (of a specific variable) should have to fit the data instead of using splines. This implies that a graphical interpretation of predicted values is the only option. Therefore, since most of the relationships were not incompatible with a linear fit on the ln-transformed PFAS, linear regression coefficient (β) and 95% Confidence Intervals (CIs) were reported. 

GAM analyses have also been stratified according to gender, and an interaction term between gender and ln-PFAS was also added to the main models.

Associations between the mixture of the three most represented PFAS (PFOA, PFOS, and PFHxS) and each outcome were evaluated applying a Weighted Quantile Sum (WQS) regression model [13]. This approach summarizes the overall exposure creating a weighted linear index of correlated predictors in terms of quantiles (quartiles in this work), weighted for the strength of their association with the response. An important toxic PFAS in the mixture was identified when its weight was ≥1/p = 1/3 = 0.33, meaning that it exceeded the case of uniform weights. Coefficients and their relative 95% CIs were estimated employing the “gWQS” R package (60% of data were used as an estimation set; 100 Bootstrap samples were computed). All analyses were fully adjusted for the established set of covariates.

A *p*-value of <0.05 was considered statistically significant. The statistical software STATA/SE version 13.0 (Stata Corp LP, College Station, TX, USA) and R (R Development Core Team 2010, R Foundation for Statistical Computing, Vienna, Austria. ISBN 3-900051-07-0, URL: http://www.R-project.org/, accessed on 15 November 2021) were used for statistical analyses. 

## 3. Results

### 3.1. General Characteristics of the Study Population and PFAS Internal Dose

The general characteristics of children and adolescents who participated in the study are reported in Table 1 and Table 2. The mean age was 16.2 years for adolescents and 9.4 years for children. The study population included 3411 (51%) males and 3258 (49%) females among adolescents and 1356 (50%) males and 1337 (50%) females among children. Most participants were living in the red area from birth (*n*= 6539, 98% of adolescents, and *n* = 2669, 99% of children), therefore being exposed to PFAS via contaminated drinking water from birth. Percentiles for all PFAS are presented as supplementary material, Table S2. Serum PFOA levels were an order of magnitude higher than PFOS and PFHxS, with a mean (SD) of 59.7 (53.0) ng/mL in male adolescents and a mean of 43.2 (38.6) ng/mL in female counterparts (*p* < 0.001), and 27.6 (22.7) ng/mL and 24.8 (20.2) ng/mL in male and female children, respectively (*p* < 0.001). Similar to other studies, PFOA, PFOS and PFHxS concentration levels were higher in male adolescents compared to females. The gender difference in serum PFOA, PFOS and PFHxS concentrations was more pronounced in the adolescent age group. PFAS concentrations were positively correlated with one another with the highest correlation observed between PFOA and PFHxS with a Spearman correlation coefficient of 0.93 and 0.89 in adolescents and children, respectively. PFHxS and PFNA were the least correlated at 0.34 among adolescents and at 0.20 among children (supplementary material, Table S3). The distribution and frequencies of the covariates among male and female adolescents and children are shown in supplementary material, Table S4.

### 3.2. Generalized Additive Models (GAM)

The regression coefficient for each PFAS (β) and 95% Confidence Intervals (CIs) estimated using generalized additive models (GAMs) are reported in the next three paragraphs, for lipid serum, blood pressure and BMI z-score, respectively. Full models showing the effect of all covariates included in the models of the association between PFOS and TC, SBP and BMI are presented in Tables S5–S7.

#### 3.2.1. Serum Lipids

Among adolescents, differences were observed in the lipid profiles between males and females, with the latter showing higher levels of TC, HDL-C, and LDL-C, and lower levels of triglycerides (Table 1). Among children, gender differences on lipid profile were limited to HDL-C (higher in males) and triglycerides (higher in females) (Table 2).

Table 3 displays the association between single PFAS and serum lipids. Among adolescents, significant associations were detected between all investigated PFAS and TC, LDL-C, and to a lesser extent HDL-C in the multivariable model adjusting for various confounders, and the associations remained significant after categorizing PFAS. Among children, PFOS and PFNA had significant associations with TC, LDL-C and HDL-C, while PFOA and PFHxS had significant associations with HDL-C only. The magnitude of the associations, measured by the increase in each lipid biomarker for a ln-increase in the serum concentration of PFAS, varied according to the PFAS congener and the age group. With regard to TC, LDL-C, and HDL-C, the largest effects were observed for PFOS; moreover, these effects were larger in children compared to adolescents (Table 3).

In adolescents, PFAS/lipids associations were not modified by gender except for HDL-C with effect significantly higher in girls (supplementary material, Table S8). In children, the effect of PFOS and PFHxS on TC and LDL-C was significantly higher in girls than in boys (supplementary material, Table S9).

#### 3.2.2. Blood Pressure

No statistically significant associations were observed between any of the investigated four PFAS concentrations and blood pressure (Table 4), neither in adolescents nor in children, after adjustment for covariates when these cardiometabolic traits were modeled as continuous outcomes in the regression models. The results stratified by gender are shown in supplementary Tables S10 (adolescents) and S11 (children).

#### 3.2.3. BMI Z-Score

Higher serum concentrations of PFAS, particularly PFOS, were associated with lower BMI z-score, among adolescents, with associations more pronounced in females (p-interaction < 0.01 for all PFAS). Greater negative effects were seen among children, with the strongest effect for PFOS, and with no gender-PFAS significant interactions (Table 5).

### 3.3. Weighted Quantile Sum (WQS) Models

In the WQS analysis, the weighted quantile sum indices for mixtures showed a positive association with TC, HDL-C and LDL-C both in adolescents and in children (Table 6).

A one-quartile increase in weighted quantile sum index was associated with a 2.35 mg/dL (CI: 1.47;3.23) increase in TC in adolescents and a 2.43 mg/dL (CI: 1.24;3.62) increase in TC in children. Quartile increases in the WQS index were associated with an increase in the HDL-C concentration of 0.68 mg/dL (CI: 0.34;1.01) and 1.14 mg/dL (0.58;1.70) in adolescents and children, respectively. Finally, for every quartile increase in the WQS index, the level of LDL-C increased of 1.79 mg/dL (CI: 0.99;2.59) in adolescents and 1.49 mg/dL (CI: 0.41;2.58) in children. A decrease in BMI z-score in both groups was also associated with a quartile increase in the PFAS mixture (−0.08 mmHg with CI: −0.12;−0.04 in adolescents and −0.1 mmHg with CI: −0.17;−0.03 in children). The level of triglycerides increased of 0.01 mg/dL (CI: 0;0.02), only in adolescents. No significant effect was detected between the mixture of chemicals and TG, SBP and DBP.

The WQS weights are also shown in Table 6. In adolescents, we identified PFOS as contributing the most to the weighted quantile sum index for TC (weight: 0.83), HDL-C (weight: 0.99), LDL-C (weight: 0.54) and BMI (weight: 0.78); PFOA and PFHxS had the lowest weights for both TC, HDL-C and BMI, but PFHxS was identified as a chemical of concern for LDL-C (weight: 0.38). For TG, the PFAS with higher weight in the mixture was PFOA (weight: 0.99). In children, PFOS showed the highest weight for all lipid parameters (weights: 0.96 for TC, 0.82 for HDL-C, 0.94 for LDL-C), while PFOA and PFHxS never exceeded the 33% threshold investigating cholesterol levels. PFOS followed by PFHxS were identified as chemicals of concern for BMI, with contributing weights of 0.51 and 0.40, respectively.

## 4. Discussion

### 4.1. Serum Lipids

In this cross-sectional study on 6669 adolescents and 2693 children exposed to high levels of PFAS through contaminated drinking water, we found a significant, non-linear association between serum concentrations of four PFAS congeners and common biomarkers of lipid metabolism. Compared to PFOA and PFHxS, PFOS and PFNA exhibited stronger associations and their effects showed a greater magnitude among children compared to adolescents. The concentration–response curves were sometimes irregular in shape, but in some instances showed a clear log-linear shape with steeper slopes at lower concentrations.

These findings are quite similar to those we previously reported in a group of more than 16,000 young adults (age 20–39 years) recruited in the Veneto Region’s health surveillance program [14]; moreover, in that sub-population, we found that PFOS had a stronger effect compared to PFOA and PFHxS and that the relationship was log-linear.

The effect size was small; however, it should be considered in light of the large population potentially exposed to low levels of PFAS and of the evidence linking childhood dyslipidemia to accelerated atherosclerosis, which prompted some scientific associations to release specific guidelines for cardiovascular risk reduction in children and adolescents [15]. A number of studies examined the association between serum PFAS and serum lipids in humans [16] but only twelve involved children or adolescents [7,8,9,17,18,19,20,21,22,23,24,25]. Even though the number of studies is relatively small, the evidence quite consistently shows a positive association between serum PFAS (mainly PFOA and PFOS) and serum cholesterol in children and adolescents, and the present study further contributes to strengthen that evidence. In those young age groups, it may be easier to observe adverse effects of environmental contaminants on the lipid metabolism since the impact of lifestyle habits has been more limited compared to adults. Most of the abovementioned studies found significant associations between one or more PFAS congeners and lipid biomarkers in children or adolescents, with few exceptions [9,22]. The most consistent effects observed across different studies regarded TC and LDL-C, while fewer studies reported a positive association with HDL-C [17,21,22,25]. The results varied between studies in terms of the magnitude and strength of the observed associations and also regarding the involved congeners. Such heterogeneity may be at least in part attributed to the limited sample size of most studies (a few hundred individuals in most instances) and also to differences in the population ages and exposure levels. All these differences render the available studies difficult to compare with each other. The study by Frisbee and colleagues [17] resembles more closely the present one, as it was based on more than 12,000 individuals aged 1–17 years from a community highly exposed to PFAS through drinking water in the Mid-Ohio valley, USA. With a cross-sectional design, the authors showed a significant log-linear association of both serum PFOA and PFOS with TC and LDL-C and of PFOS with HDL-C. With regard to TC and LDL-C, the magnitude of the association was greater for PFOS. Altogether, these results are similar to what we found in the Veneto Region’s exposed population.

Although the literature consistently shows an association between serum PFAS concentrations and serum cholesterol levels, it is not clear whether this association is causal or not. To date, most epidemiological studies, including ours, have been cross-sectional, thus limiting any causal inference. Cross-sectional studies are subject to bias, such as reverse causation or confounding, as has also been suggested for the PFAS–lipids associations. In particular, a confounding effect due to individual variations in the enterohepatic cycling of PFOS/PFOA and bile acids has been hypothesized [26], and the European Food Safety Authority has recently reviewed its former decision to consider the increase in serum cholesterol as one of the critical effects of PFAS exposure [16,27]. As regards children and adolescents, only four cohort studies have been conducted so far on the associations between maternal serum PFAS during pregnancy and offspring serum lipids later in life [8,9,24,25]. The results were inconsistent, with some studies reporting some positive associations [8,24,25] and others not [9]. Irrespective of this, even a well-conducted cohort study may provide limited evidence on the causal link between serum PFAS and cholesterol if the biomarker of exposure (i.e., serum PFAS) is determined only once and the outcome is assessed after a long delay, since cholesterol levels may be sensitive to modifications of exposures and the PFAS effect on cholesterol may be transient and reversible. The latter hypothesis is suggested by a study from the Mid-Ohio valley community, which showed a greater decrease in serum cholesterol in association with the decrease in serum PFAS [28]. Further studies based on repeated measures of PFAS and lipids levels in the same individuals may contribute to shed light on the intricated issue of the PFAS–lipids association.

We observed some significant gender differences in the associations between PFAS and serum lipids, and those gender differences varied according to age range. The effect of PFOA, PFOS, and PFHxS on HDL-C was higher in female adolescents, while the effect of PFOS and PFHxS on TC and LDL-C was higher in female children. In our previous study on the young adult population, we observed that the associations between PFAS and HDL-C were statistically significant only in females [14]. Among other studies, only a few assessed gender effect modification, with variable findings: in the Mid-Ohio valley community, larger effects of PFOA and PFOS on TC and LDL-C were found in boys compared to girls [17], whereas in the study by Mora et al. [25] mid-childhood serum PFOA, PFOS and PFDeA were associated with a larger increase in TC and LDL-C among girls. Another study [9] did not find any significant gender differences. Two studies recruited only females and provided contradictory findings: in a cross-sectional study of girls aged 6–8 years, Fassler et al. [22] showed a positive association of PFOA with HDL, while the cohort study by Maisonet et al. [8] showed a positive association of prenatal PFOA (limited to the lower tertile of the distribution) with TC and LDL-C in followed-up daughters at the ages of 7 and 15 years.

Overall, the results of the combined effects of the PFAS mixture support findings under the single-chemical linear regressions: higher concentrations of PFAS are associated with higher levels of serum lipid parameters, especially TC and LDL-C.

Altogether, our findings and the available literature indicate the existence of a very complex interplay between PFAS exposure and individual characteristics such as gender and phase of the life course, possibly related to the variation in the internal doses across genders [29,30] and ages and to the effect of sex hormones on lipid metabolism [31,32]. In this regard, mechanistic evidence is accruing on a pro-estrogenic and anti-androgenic effect of PFAS [33,34]. Further mechanistic studies are needed to understand whether these hormonal perturbations are linked to alterations of lipid metabolism.

### 4.2. Blood Pressure

To our knowledge, only one study has, by now, found a positive association between hypertension and PFAS serum levels in an adolescent population, while they did not find any association of BP as a continuous variable and PFAS concentration [7]. Other studies, with both cross-sectional and longitudinal design, did not find any significant association [9,35]. This evidence is supported by our results, for both adolescents and children, using both GAM models to investigate the single PFAS exposure and WQS regression models to assess mixture effect on systolic and diastolic blood pressure.

### 4.3. BMI

Our results, both single- and multi-pollutants, indicate that higher serum concentrations of PFAS, particularly PFOS, were associated with lower BMI z-score during both childhood and adolescence, with a significant effect of gender in the latter. In the literature, the outcome of PFAS on adiposity are inconsistent and often contradictory, with studies reporting both positive and negative associations, or no association, as discussed below. Few cross-sectional studies have examined the relationship between childhood/adolescence exposure to PFAS and BMI/overweight showing inconsistent findings, although the association between prenatal PFOA and BMI was mostly inverse [36,37] or null [38,39], except for one study [24]. In particular, analysis of the BMI trajectory in infants up to 12 years old showed that BMI zenith was lower in magnitude in the highest tertile of PFOA [37]. In children aged 3 to 11 years old, PFHxS was negatively associated with weight-for-age and BMI z-score, but only in males [40]. Recently, Pinney et al. (2019) investigated the relationship between serum PFOA in girls aged 6–8 years and longitudinal changes in adiposity at age 6–18 years. The authors reported an inverse association of PFOA level with BMI z-score, but declining with age [41], which is not in agreement with our data, although the time window of our study is tighter and the impact of puberty could have unmasked this association in the Pinney study. In another cohort study on girls aged 6 to 8 years, increasing serum PFOA concentrations were associated with decreased BMIz and fat mass percent [22].

These findings may support evidence of continued negative effects on weight by prenatal PFAS exposures. Few studies have investigated the association between PFAS and adiposity during puberty, with conflicting results according to sex, timing of exposure, type of study, lifestyle factors (such as physical activity, diet, sleep, and stress) and levels of exposure. Koshy et al. found no association with overweight in American adolescents exposed to PFHxS, PFOA, PFOS, PFNA, PFDA [20], whereas a Swedish prospective birth cohort study showed a positive association of PFOS and PFOA exposure with overweight/obesity [42]. A large multicenter prospective cohort study (the European Youth Heart Study) showed that childhood exposure to PFOS and PFOA predicted adiposity at 15 and 21 years of age [10]. A very recent cross-sectional study [7] found a positive association between PFHxS and PFHpS serum levels with obesity in Norwegian adolescents; however, this association was not linear and there was no positive association with other PFAS.

In agreement with the literature, we also reported a greater magnitude for the association in girls than boys. Indeed, growing evidence suggests that the association of early-life exposure to certain environmental toxicants with placental functions and risk of disease later in life may vary by child sex [43]. One possible mechanism to explain gender differences in the association of PFAS with childhood or adolescent adiposity could be related to increased cortisol levels associated with PFAS exposure [44] and/or associated to placental epigenetic processes with sex-specific effects, as observed for maternal stress [45]. As serum levels of androgens and gonadotropins differ between sexes during mini-puberty in early childhood [46], it cannot be excluded that the observed associations may be influenced by sex differences in hormones. In vitro studies [31,32] demonstrated that PFAS have estrogenic and antiandrogenic activities, but the in vivo consequences to hormonal interference by PFAS might also differ to receptor sensitivity differences between the two sexes, due to the physiological homeostasis of sex steroids [47,48]. In addition, PFAS can inhibit 11-β hydroxysteroid dehydrogenase 2 with subsequent increases in glucocorticoid concentrations [49], leading to alterations in placental development and function, and impairment of fetal growth [47]. However, the inverse association between PFAS and BMI could be confounded by pubertal status, and particularly menstruation status among girls. Indeed, young women who already had menarche are expected to have lower PFAS levels due to menstruation loss and higher age-matched BMI.

Since obesity is a complex disease with multifactorial etiology, differences between studies and populations may be attributable to different genetic and environmental factors, study designs, concentrations of PFAS, or the timing and method of adiposity measurements. In particular, the differences between cross-sectional and longitudinal studies might be explained by reverse causation associated with the expanded distribution volumes in obese compared to lean children [39]. For instance, in the longitudinal study by Liu et al. (2020), prenatal PFAS concentrations were overall weakly correlated with postnatal PFAS concentrations [48]. In addition, some of the point estimates for postnatal PFAS concentration were negative, whereas their prenatal counterparts were positive. In our study, the use of a single serum measure may not fully reflect past exposure, and this exposure measure does not allow for assessment of prenatal exposures. However, these substances have long half-lives in humans, which may be upwards of 20 years [30], so exposure misclassification is less likely. Moreover, it was suggested that PFAS exposure during foetal life has a minor impact on childhood anthropometry and weight than PFAS exposure from the environment where children grow up [49].

The mechanism through which PFAS may interfere with childhood and adolescence adiposity remains unclear. PFAS were found to activate peroxisome proliferator activated receptor-alpha (PPARα) or (PPARγ), which are essential for the regulation of insulin signaling, and glucose and lipid metabolism.

In addition, cortisol and sex hormone receptors, together with thyroid metabolism, have been shown to represent targets of PFAS [50,51,52]. Since thyroid hormones play a crucial role in normal growth and development, altered thyroid function can affect early-life growth and adiposity during critical periods of development. There is a possibility that the effect of PFAS on early-life growth can be mediated by thyroid hormone disruption. Finally, the wide variety of pathways altered by PFAS may also reflect the inconsistencies across studies among different PFAS molecules, since each compound may have different affinities and magnitudes of effect depending on the targeted pathway and the relative outcome.

### 4.4. Strengths and Limitations

The strengths of this study include the large population, the accurate measurement of internal exposure to PFAS, and information on several anthropometric, lifestyle, and clinical variables, which allowed for the adjustment of many possible confounders. We were able to model concentration–response curves of PFOA over a wide range of internal doses, covering both background and high exposure levels. The assessment of the concentration–response relation over the entire range of exposure makes our results valuable also for other populations with only background exposure. Moreover, we systematically evaluated gender-specific associations. Our study suffers from several limitations, however. The cross-sectional design precludes evaluation of the temporal relationship between exposure and outcome and thus results may be affected by reverse causation. Moreover, we cannot exclude covariate misclassification and residual confounding due to unmeasured risk factors such as sleep and stress. Another important limitation is that in most subjects we relied on a single blood pressure measurement, which may have led to a non-differential misclassification of this outcome due to random error.

## 5. Conclusions

Our cross-sectional study in children and adolescents supports a positive association between serum PFAS concentration and serum lipids, stronger for PFOS and PFNA and with a greater magnitude among children compared to adolescents, and a negative association of PFAS with BMI. Additional studies are needed to verify these findings, and long-term studies that examine growth trajectories from birth through childhood and adolescence would help elucidate the associations between prenatal PFAS exposure and growth. Gender differences may come from the limitations of research design, but there may also be a sex-specific dynamic to PFAS exposure.

## Figures and Tables

**Table 1:** Characteristics of the ADOLESCENTS included in the study population (*n* = 6669), stratified by gender.

PFAS/Outcomes	Total			Males (*n* = 3411)			Females (*n* = 3258)		
	Mean (SD)	Min–Max	Median (Q1–Q3)	Mean (SD)	Min–Max	Median (Q1–Q3)	Mean (SD)	Min–Max	Median (Q1–Q3)
**PFAS**									
PFOA	51.6 (47.2)	0.4–599.3	38.9 (20.1–68.8)	59.7 (53.0)	0.4–599.3	45.2 (24.6–79.3)	43.2 (38.6)	0.4–477.3	33.3 (16.5–58.7)
PFOS	4.1 (3.5)	0.4–86.8	3.3 (2.2–4.9)	4.5 (3.6)	0.4–69.1	3.6 (2.5–5.3)	3.7 (3.2)	0.4–86.8	3.0 (2.0–4.4)
PFHxS	3.6 (2.9)	0.4–27.2	2.8 (1.6–4.8)	4.2 (3.2)	0.4–27.2	3.4 (2.0–5.6)	3.0 (2.3)	0.4–21.6	2.4 (1.3–3.9)
PFNA	0.5 (0.3)	0.4–3.7	0.35 (0.35–0.6)	0.5 (0.2)	0.4–3.7	0.4 (0.4–0.6)	0.5 (0.3)	0.4–3.2	0.4 (0.4–0.6)
**OUTCOMES ^1^**									
TOTAL CHOLESTEROL	150.5 (27.1)	60–294	148 (132–166)	145.0 (26)	77–290	143 (127–161)	156.2 (27.1)	60–294	153 (138–172)
HDL CHOLESTEROL	54.2 (11.9)	19–124	53 (46–61)	50.1 (10.2)	19–99	49 (43–56)	58.4 (12.0)	24–124	57 (50–66)
LDL CHOLESTEROL	79.5 (23.0)	0–243	77 (64–93)	76.7 (23.0)	0–243	75 (61–90)	82.4 (22.7)	6–211	80 (67–95)
TRYGLICERIDES	84.9 (50.3)	17–699	71 (54–100)	92.3 (58.2)	17–699	76 (56–110)	77.0 (38.8)	17–505	67 (52–90)
BMI z-score	0.3 (1.2)	−4.7–4.5	0.26 (−0.5–1.0)	0.3 (1.1)	−4.73–4.26	0.28 (-0.38–1.03)	0.25 (1.2)	−3.73–4.54	0.2 (-0.6–1.1)
SYSTOLIC BP	114.5 (13.9)	70–190	115 (105–120)	117.9 (14.1)	70–190	120 (110–129)	111.0 (12.8)	70–180	110 (100–120)
DIASTOLIC BP	66.9 (9.6)	30–124.5	70 (60–71)	67.5 (10.0)	30–105	70 (60–75)	66.2 (9.1)	40–124.5	65 (60–70)

^1^ Reference ranges for lipids: TC < 190 mg/dL, LDL-C < 115 mg/dL, HDL-C > 40 mg/dL, TG < 150 mg/dL.

**Table 2:** Characteristics of the CHILDREN included in the study population (*n* = 2693), stratified by gender.

PFAS/Outcomes	Total			Males (*n* = 1356)			Females (*n* = 1337)		
	Mean (SD)	Min–Max	Median (Q1–Q3)	Mean (SD)	Min–Max	Median (Q1–Q3)	Mean (SD)	Min–Max	Median (Q1–Q3)
**PFAS**									
PFOA	26.2 (21.5)	0.4–316.3	20.9 (12.9–33.5)	27.6 (22.7)	0.4–316.3	22.5 (13.5–35.82)	24.8 (20.2)	0.4–209.3	19.8 (12.2–31.0)
PFOS	2.6 (2.5)	0.4–96	2.2 (1.6–3.0)	2.6 (3.0)	0.4–96.0	2.2 (1.6–3.1)	2.5 (1.9)	0.4–39.0	2.1 (1.5–2.9)
PFHxS	2.2 (1.5)	0.4–14.6	1.9 (1.2–2.8)	2.3 (1.6)	0.4–14.6	2.0 (1.3–3.0)	2.1 (1.4)	0.4–13.8	1.8 (1.1–2.6)
PFNA	0.4 (0.2)	0.4–3.1	0.4 (0.4–0.5)	0.4 (0.1)	0.4–1.2	0.4 (0.4–0.5)	0.4 (0.2)	04–3.1	0.4 (0.4–0.5)
**OUTCOMES ^1^**									
TOTAL CHOLESTEROL	160.8 (27.6)	77–389	159 (143–176.0)	161.0 (28.0)	80–357	159 (142–176.0)	160.6 (27.1)	77–389	158 (143–176.0)
HDL CHOLESTEROL	59.5 (12.1)	21–111	59 (51–67.0)	60.7 (12.5)	24–107	60 (52–69.0)	58.3 (11.6)	21–111	58 (50–66.0)
LDL CHOLESTEROL	88.5 (24.8)	16–323	86 (72–102.0)	88.2 (25.1)	19–271	86 (71–102.0)	88.9 (24.5)	16–323	87 (73–102.5)
TRYGLICERIDES	64.0 (31.8)	16–383	57 (43–75.0)	60.9 (31.1)	16–303	54 (40–71.5)	67.1 (32.3)	22–383	60 (46–78.0)
BMI z-score	0.7 (1.3)	−5.7–5.6	0.7 (−0.2–1.7)	0.7 (1.2)	−3.87–5.61	0.7 (−0.2–1.6)	0.7 (1.4)	−5.68–4.71	0.7 (−0.2–1.7)
SYSTOLIC BP	100.0 (11.1)	70–160	100 (90–108)	99.7 (11.1)	70–160	100 (90–108)	100.2 (11.1)	70–142	100 (90–109)
DIASTOLIC BP	62.0 (8.3)	45–100	60 (57–70)	62 (8.3)	45–100	60 (56–70)	62.0 (8.2)	45–93	60 (57–70)

^1^ Reference ranges for lipids: TC < 190 mg/dL, LDL-C < 115 mg/dL, HDL-C > 40 mg/dL, TG < 150 mg/dL.

**Table 3:** Association between ln-PFAS (ln ng/mL) and serum lipids (mg/dL) from GAM models: adjusted β* coefficients and 95% Confidence Intervals (CIs).

PFAS	Adolescents				Children			
	TC	HDL	LDL	TRIGLYCERIDES	TC	HDL	LDL	TRIGLYCERIDES
	Coef (95% CI)	Coef (95% CI)	Coef (95% CI)	Coef (95% CI)	Coef (95% CI)	Coef (95% CI)	Coef (95% CI)	Coef (95% CI)
ln_pfoa	1.05 (0.31, 1.80)	−0.17 (−0.47, 0.14)	1.03 (0.39, 1.66)	0.01 (0.00, 0.03)	0.85 (−0.44, 2.14)	0.64 (0.09, 1.19)	0.17 (−0.98, 1.32)	0 (−0.02, 0.02)
II Q	1.18 (−0.60, 2.96)	−0.02 (−0.74, 0.71)	1.19 (−0.33, 2.71)	0.01 (−0.02, 0.04)	0.45 (−2.43, 3.33)	0.71 (−0.51, 1.94)	−0.23 (−2.8, 2.34)	−0.01 (−0.05, 0.04)
III Q	1.68 (−0.18, 3.55)	0.10 (−0.67, 0.86)	1.55 (−0.05, 3.15)	0.00 (−0.03, 0.04)	0.31 (−2.59, 3.21)	1.35 (0.12, 2.58)	−1.09 (−3.68, 1.5)	−0.01 (−0.05, 0.03)
IV Q	3.20 (1.20, 5.20)	−0.30 (−1.12, 0.52)	2.99 (1.27, 4.70)	0.04 (0.01, 0.07)	2.97 (0.02, 5.93)	1.46 (0.20, 2.71)	1.58 (−1.06, 4.21)	−0.01 (−0.06, 0.03)
ln_pfos	3.32 (2.20, 4.45)	1.17 (0.71, 1.63)	2.66 (1.70, 3.62)	−0.02 (−0.04, 0.00)	6.22 (4.32, 8.13)	1.91 (1.1, 2.73)	4.52 (2.8, 6.23)	−0.01 (−0.04, 0.02)
II Q	3.75 (1.98, 5.52)	1.02 (0.29, 1.74)	3.03 (1.51, 4.54)	−0.01 (−0.04, 0.02)	3.80 (1.05, 6.56)	2.32 (1.14, 3.49)	2.08 (−0.39, 4.55)	−0.02 (−0.07, 0.02)
III Q	4.10 (2.26, 5.95)	1.30 (0.54, 2.05)	3.11 (1.53, 4.69)	−0.02 (−0.05, 0.01)	5.82 (2.96, 8.68)	2.35 (1.13, 3.57)	3.52 (0.96, 6.09)	0.01 (−0.04, 0.05)
IV Q	5.84 (3.88, 7.79)	1.83 (1.04, 2.63)	4.63 (2.96, 6.31)	−0.03 (−0.06, 0.01)	8.34 (5.51, 11.17)	2.99 (1.78, 4.20)	5.83 (3.28, 8.39)	−0.04 (−0.08, 0)
ln_pfhxs	1.49 (0.60, 2.37)	−0.05 (−0.41, 0.31)	1.44 (0.68, 2.19)	0.01 (−0.01, 0.02)	1.30 (−0.28, 2.88)	0.8 (0.12, 1.47)	0.54 (−0.87, 1.96)	−0.01 (−0.03, 0.01)
II Q	1.96 (0.20, 3.73)	−0.16 (−0.88, 0.56)	2.03 (0.52, 3.55)	0.01 (−0.02, 0.04)	−1.04 (−3.84, 1.76)	0.46 (−0.73, 1.65)	−1.70 (−4.19, 0.8)	0 (−0.04, 0.04)
III Q	1.72 (-0.10, 3.54)	0.14 (−0.60, 0.88)	1.60 (0.05, 3.16)	0.00 (−0.03, 0.03)	0.56 (−2.35, 3.46)	1.68 (0.44, 2.91)	−1.22 (−3.81, 1.38)	0 (−0.04, 0.04)
IV Q	3.80 (1.83, 5.77)	0.07 (−0.74, 0.87)	3.65 (1.97, 5.33)	0.02 (−0.02, 0.05)	1.95 (−0.99, 4.89)	1.32 (0.07, 2.56)	0.76 (−1.86, 3.39)	−0.02 (−0.07, 0.02)
PFNA low	3.43 (1.91, 4.95)	1.07 (0.45, 1.69)	2.35 (1.05, 3.65)	0.00 (−0.02, 0.03)	2.95 (0.39, 5.50)	1.31 (0.22, 2.40)	1.58 (−0.71, 3.87)	−0.01 (−0.05, 0.03)
PFNA medium	3.58 (1.83, 5.33)	1.45 (0.73, 2.17)	3.04 (1.54, 4.54)	−0.03 (−0.06, 0.00)	7.53 (3.81, 11.25)	1.8 (0.21, 3.38)	6.06 (2.73, 9.39)	−0.02 (−0.07, 0.04)

**Table 4:** Association between PFAS (ln ng/mL) and blood pressure (mmHg) from GAM models: adjusted β* coefficients and 95% Confidence Intervals (CIs).

PFAS	Adolescents		Children	
	SYSTOLIC BP	DIASTOLIC BP	SYSTOLIC BP	DIASTOLIC BP
	Coef (95% CI)	Coef (95% CI)	Coef (95% CI)	Coef (95% CI)
ln_pfoa	−0.16 (−0.53, 0.20)	−0.11 (−0.37, 0.15)	−0.51 (−1.02, −0.01)	0.16 (−0.23, 0.54)
II Q	−0.44 (−1.31, 0.43)	−0.23 (−0.84, 0.39)	−0.08 (−1.20, 1.05)	0.58 (−0.28, 1.44)
III Q	−1.01 (−1.92, −0.10)	−0.28 (−0.93, 0.36)	−0.22 (−1.35, 0.91)	0.37 (−0.5, 1.24)
IV Q	−0.44 (−1.42, 0.54)	−0.08 (−0.77, 0.61)	−0.98 (−2.14, 0.18)	0.68 (−0.21, 1.57)
ln_pfos	−0.47 (−1.02, 0.08)	−0.44 (−0.82, −0.05)	−0.42 (−1.18, 0.33)	0.03 (−0.54, 0.61)
II Q	−0.67 (−1.54, 0.2)	−0.54 (−1.15, 0.08)	−0.13 (−1.22, 0.95)	0.67 (−0.16, 1.5)
III Q	−0.96 (−1.87, −0.06)	−0.66 (−1.30, −0.02)	0.18 (−0.95, 1.31)	0.91 (0.05, 1.77)
IV Q	−1.34 (−2.3, −0.38)	−0.78 (−1.45, −0.10)	−0.8 (−1.92, 0.33)	−0.1 (−0.95, 0.75)
ln_PFHxS	−0.22 (−0.65, 0.21)	−0.15 (−0.45, 0.16)	−0.68 (−1.3, −0.06)	0.15 (−0.33, 0.62)
II Q	−0.34 (−1.21, 0.52)	0.02 (−0.59, 0.63)	−0.53 (−1.62, 0.57)	0.69 (−0.15, 1.54)
III Q	−0.71 (−1.6, 0.18)	−0.17 (−0.79, 0.46)	−1.38 (−2.52, −0.24)	0.19 (−0.68, 1.06)
IV Q	−0.37 (−1.33, 0.59)	−0.29 (−0.97, 0.39)	−1.12 (−2.28, 0.03)	0.3 (−0.58, 1.19)
PFNA low	−0.58 (−1.32, 0.17)	0.38 (−0.15, 0.90)	−0.45 (−1.45, 0.56)	0.32 (−0.45, 1.08)
PFNA medium	−0.33 (−1.19, 0.53)	0.07 (−0.54, 0.68)	−0.23 (−1.69, 1.23)	−0.91 (−2.03, 0.2)

**Table 5:** Association between PFAS (ln ng/mL) and BMI z-scores from GAM models: adjusted β* coefficients and 95% Confidence Intervals (CIs), stratified by gender.

PFAS	BMI z-Score—Adolescents			BMI z-Score—Children		
	TOTAL	MALES	FEMALES	TOTAL	MALES	FEMALES
	Coef (95% CI)	Coef (95% CI)	Coef (95% CI)	Coef (95% CI)	Coef (95% CI)	Coef (95% CI)
ln_pfoa	−0.03 (−0.06, 0.01)	0 (−0.04, 0.04)	−0.07 (−0.11, −0.02)	−0.08 (−0.14, −0.01)	−0.07 (−0.15, 0.01)	−0.08 (−0.18, 0.02)
II Q	−0.10 (−0.18, −0.02)	−0.10 (−0.21, 0.01)	−0.09 (−0.2, 0.02)	0.03 (−0.11, 0.17)	0.20 (0.01, 0.39)	−0.10 (−0.32, 0.11)
III Q	−0.10 (−0.18, −0.02)	−0.06 (−0.17, 0.05)	−0.11 (−0.23, 0)	−0.11 (−0.25, 0.03)	0.01 (−0.18, 0.2)	−0.20 (−0.41, 0.02)
IV Q	−0.06 (−0.14, 0.03)	−0.03 (−0.14, 0.09)	−0.16 (−0.29, −0.02)	−0.17 (−0.32, −0.03)	−0.09 (−0.27, 0.1)	−0.25 (−0.48, −0.02)
ln_pfos	−0.11 (−0.16, −0.06)	−0.10 (−0.17, −0.04)	−0.16 (−0.23, −0.09)	−0.27 (−0.36, −0.17)	−0.24 (−0.36, −0.11)	−0.31 (−0.45, −0.17)
II Q	−0.14 (−0.22, −0.07)	−0.13 (−0.24, −0.02)	−0.11 (−0.22, 0)	−0.15 (−0.28, −0.01)	−0.01 (−0.19, 0.17)	−0.29 (−0.5, −0.09)
III Q	−0.16 (−0.24, −0.08)	−0.14 (−0.26, −0.03)	−0.20 (−0.32, −0.09)	−0.25 (−0.39, −0.11)	−0.16 (−0.35, 0.03)	−0.33 (−0.55, −0.12)
IV Q	−0.19 (−0.27, −0.1)	−0.16 (−0.28, −0.05)	−0.26 (−0.39, −0.13)	−0.35 (−0.49, −0.21)	−0.3 (−0.48, −0.12)	−0.42 (−0.63, −0.2)
ln_PFHxS	0.03 (−0.01, 0.07)	0.06 (0.01, 0.11)	−0.03 (−0.09, 0.03)	−0.13 (−0.21, −0.05)	−0.11 (−0.21, −0.01)	−0.14 (−0.26, −0.03)
II Q	−0.08 (−0.15, 0)	0 (−0.12, 0.11)	−0.11 (−0.22, 0)	0.06 (−0.08, 0.2)	0.12 (−0.06, 0.31)	0 (−0.2, 0.21)
III Q	0.01 (−0.07, 0.09)	0.09 (−0.02, 0.2)	−0.04 (−0.15, 0.08)	−0.20 (−0.34, −0.06)	−0.11 (−0.3, 0.08)	−0.25 (−0.47, −0.04)
IV Q	0.03 (−0.05, 0.12)	0.12 (0, 0.23)	−0.11 (−0.25, 0.02)	−0.18 (−0.32, −0.03)	−0.12 (−0.31, 0.07)	−0.23 (−0.46, −0.01)
PFNA low	−0.02 (−0.08, 0.05)	0.03 (−0.06, 0.11)	−0.1 (−0.2, 0)	−0.25 (−0.38, −0.13)	−0.28 (−0.44, −0.13)	−0.21 (−0.41, −0.01)
PFNA medium	−0.04 (−0.11, 0.04)	0 (−0.1, 0.1)	−0.11 (−0.23, 0)	−0.36 (−0.54, −0.18)	−0.37 (−0.62, −0.12)	−0.35 (−0.62, −0.08)

**Table 6:** Associations between WQS regression index and serum lipids (mg/dL), blood pressure (mmHg) and BMI. WQS regression model weights of each PFAS component, for each outcome.

**ADOLESCENTS**							
**Outcome**	**TC**	**HDL-C**	**LDL-C**	**TG**	**BMI**	**SBP**	**DBP**
β* **95% CI**	**2.35** [1.47, 3.23]	**0.68** [0.34, 1.01]	**1.79** [0.99, 2.59]	**0.01** [0, 0.02]	**−0.08** [−0.12, −0.04]	−0.04 [−0.46, 0.37]	−0.05 [−0.33, 0.24]
**PFAS**	**Weights for each outcome**						
*PFOA*	0.03	0.00	0.08	**0.99**	0.22	**0.57**	0.06
*PFOS*	**0.83**	**0.99**	**0.54**	0.00	**0.78**	0.00	0.00
*PFHxS*	0.14	0.01	**0.38**	0.01	0.00	**0.43**	**0.94**
**CHILDREN**							
**Outcome**	**TC**	**HDL-C**	**LDL-C**	**TG**	**BMI**	**SBP**	**DBP**
β* **95% CI**	**2.43** [1.24, 3.62]	**1.14** [0.58, 1.70]	**1.49** [0.41, 2.58]	−0.01 [−0.03, 0.01]	**−0.1** [−0.17, −0.03]	−0.38 [−0.87, 0.1]	0.19 [−0.22, 0.61]
**PFAS**	**Weigths for each outcome**						
*PFOA*	0.03	0.09	0.03	**0.52**	0.09	0.01	**0.61**
*PFOS*	**0.96**	**0.82**	**0.94**	**0.40**	**0.51**	**0.92**	0.32
*PFHxS*	0.01	0.09	0.03	0.08	**0.40**	0.06	0.07

*β represents the increase in cholesterol level and blood pressure associated with a quartile increase in the WQS index. In bold weights that exceed the case of uniform weights (≥(number of chemicals) − 1 = 0.33).

## Data Availability

The data are not publicly available. The data presented in this study are available on request from the corresponding author.

## Supplementary Materials

The following are available online at https://www.mdpi.com/article/10.3390/ijerph182412881/s1, Figure S1: Directed acyclic graph (DAG) for the selection of covariates, Figure S2: Flow-chart of study population, Table S1: Detection rates for all PFAS in adolescents and children. Table S2: Percentiles for all PFAS in adolescents and children, Table S3: Correlation matrix of PFAS, adolescents (a) and children (b), Table S4: Distribution and frequencies of covariates, overall and by gender. (a) Adolescents and (b) Children. Table S5: Full model association between PFOS (ng/mL) and TC (mg/dL) from GAM models: adjusted β coefficients and confidence intervals (95%CIs). Table S6: Full model association between PFOS (ng/mL) and SBP (mmHg) from GAM models: adjusted β coefficients and confidence intervals (95%CIs). Table S7: Full model association between PFOS (ng/mL) and BMI z-score, from GAM models: adjusted β coefficients and confidence intervals (95%CIs). Table S8: Association between PFAS (ng/mL) and serum lipids (mg/dL) from GAM models, stratified by gender: adjusted β* coefficients and 95% Confidence Intervals (CIs). ADOLESCENTS, Table S9: Association between PFAS (ng/mL) and serum lipids (mg/dL) from GAM models, stratified by gender: adjusted β* coefficients and 95% Confidence Intervals (CIs). CHILDREN, Table S10: Association between PFAS (ng/mL) and blood pressure (mmHg) from GAM models, stratified by gender: adjusted β* coefficients and 95% Confidence Intervals (CIs). ADOLESCENTS, Table S11: Association between PFAS (ng/mL) and blood pressure (mmHg) from GAM models, stratified by gender: adjusted β* coefficients and 95% Confidence Intervals (CIs). CHILDREN.
