# Supplementary material for: PFAS Concentrations and Cardiometabolic Traits in Highly Exposed Children and Adolescents
Source: Int J Environ Res Public Health. 2021 Dec 7;18(24):12881. doi: 10.3390/ijerph182412881 (PMC8701234; doi:10.3390/ijerph182412881)

Table S1. Detection rates for all PFAS in adolescents and children.

PFAS	% PFAS <LOQ (0.353)						
	**Adolescents**			**Children**			**Overall**
	Males	Females	Total	Males	Females	Total	
PFOA	0.03%	0.07%	0.04%	0.22%	0.07%	0.15%	0.07%
PFOS	0.23%	0.30%	0.25%	0.44%	0.90%	0.67%	0.37%
PFHxS	2.30%	2.58%	2.41%	2.80%	4.04%	3.42%	2.70%
PFNA	56.02%	56.43%	56.19%	70.28%	73.15%	71.70%	60.65%

Table S2. Percentiles for all PFAS in adolescents and children.

**Adolescents**				
**PFAS**	**Quartile**			
	1	*2*	*3*	*4*
PFOA	[0.354,20.1]	(20.1,38.9]	(38.9,68.8]	(68.8,599]
PFOS	[0.354,2.2]	(2.2,3.3]	(3.3,4.9]	(4.9,86.8]
PFHxS	[0.354,1.6]	(1.6,2.8]	(2.8,4.8]	(4.8,27.2]
	Below LOQ		*low*	*medium*
PFNA	0.35353		(0.354,0.6]	(0.6,3.7]
**Children**				
**PFAS**	**Quartile**			
	1	*2*	*3*	*4*
PFOA	[0.354,12.9]	(12.9,20.9]	(20.9,33.5]	(33.5,316]

PFOS	[0.354,1.6]	(1.6,2.2]	(2.2,3]	(3,96]
PFHxS	[0.354,1.2]	(1.2,1.9]	(1.9,2.8]	(2.8,14.6]
	Below LOQ		*low*	*medium*
PFNA	0.35353		(0.354,0.5]	(0.5,3.1]

Table S3. Correlation matrix of PFAS: adolescents (a) and children (b).

(a)

	PFOA	PFOS	PFHxS	PFNA
PFOA	1			
PFOS	0.6421	1		
PFHxS	0.9253	0.6505	1	
PFNA	0.3626	0.5986	0.3363	1

(b)

	PFOA	PFOS	PFHxS	PFNA
PFOA	1			
PFOS	0.4464	1		
PFHxS	0.8914	0.4759	1	
PFNA	0.2102	0.5444	0.2037	1

Table S4. Distribution and frequencies of covariates, overall and by gender: (a) adolescents and (b) children.

(a)

	Freq (%)		
	**TOTAL**	**MALES**	**FEMALES**
**Gender**			
M	3411 (51.15)		
F	3258 (48.85)		
**Laboratory**			
Arzignano	3364 (50.44)	1738 (50.95)	1626 (49.91)
Legnago	1727 (25.9)	890 (26.09)	837 (25.69)
San Bonifacio	1578 (23.66)	783 (22.96)	795 (24.4)
**Center**			
Lonigo	1900 (28.49)	993 (29.11)	907 (27.84)
Legnago	1727 (25.9)	890 (26.09)	837 (25.69)
San Bonifacio	1578 (23.66)	783 (22.96)	795 (24.4)
Noventa Vicentina	1464 (21.95)	745 (21.84)	719 (22.07)
**Smoke**			
NO	5365 (80.45)	2625 (76.96)	2740 (84.1)
YES	1209 (18.13)	731 (21.43)	478 (14.67)
Ex smoker	95 (1.42)	55 (1.61)	40 (1.23)
**Physical activity**			
Light	4615 (69.2)	2018 (59.16)	2597 (79.71)
Moderate	490 (7.35)	250 (7.33)	240 (7.37)

Heavy	1564 (23.45)	1143 (33.51)	421 (12.92)
**Country of birth**			
HDC	6334 (94.98)	3236 (94.87)	3098 (95.09)
HMPC	335 (5.02)	175 (5.13)	160 (4.91)
**Salt**			
Low	1818 (27.26)	850 (24.92)	968 (29.71)
Medium	4369 (65.51)	2302 (67.49)	2067 (63.44)
High	482 (7.23)	259 (7.59)	223 (6.84)
**Age**			
Mean (SD)	16.23 (1.61)	16.21 (1.61)	16.22 (1.61)
min - max	14 , 19	14 , 19	14 , 19
Median (Q1-Q3)	16 (15 , 18)	16 (15 , 18)	16 (15 , 18)
**Time-lag**			
Mean (SD)	13.12 (10.06)	13.52 (10.06)	13.31 (10.06)
min - max	1 , 34	1 , 34	1 , 34
Median (Q1-Q3)	9 (5 , 24)	10 (5 , 24)	9 (5 , 24)

(b)

	**Frequencies (%)**		
	**TOTAL**	**MALES**	**FEMALES**
**Gender**			
M	1356 (50.35)		
F	1337 (49.65)		

**Laboratory**			
Arzignano	1261 (46.83)	653 (48.16)	608 (45.47)
Legnago	698 (25.92)	341 (25.15)	357 (26.7)
San Bonifacio	734 (27.26)	362 (26.7)	372 (27.82)
**Center**			
Lonigo	699 (25.96)	362 (26.7)	337 (25.21)
Legnago	698 (25.92)	341 (25.15)	357 (26.7)
San Bonifacio	734 (27.26)	362 (26.7)	372 (27.82)
Noventa Vicentina	562 (20.87)	291 (21.46)	271 (20.27)
**Physical activity**			
Light	1494 (55.48)	614 (45.28)	880 (65.82)
Moderate	338 (12.55)	150 (11.06)	188 (14.06)
Heavy	861 (31.97)	592 (43.66)	269 (20.12)
**Country of birth**			
HDC	2644 (98.18)	1330 (98.08)	1314 (98.28)
HMPC	49 (1.82)	26 (1.92)	23 (1.72)
**Salt**			
Low	1029 (38.21)	513 (37.83)	516 (38.59)
Medium	1607 (59.67)	817 (60.25)	790 (59.09)
High	57 (2.12)	26 (1.92)	31 (2.32)
**Age**			
Mean (SD)	9.4 (0.74)	9.39 (0.74)	9.42 (0.75)
min - max	8-11	8-11	8-11

Median (Q1-Q3)	9 (9-10)	9 (9-10)	9 (9-10)
**Time-lag**			
Mean (SD)	26.75 (3.38)	26.76 (3.38)	26.75 (3.39)
min - max	9-34	9-34	22-34
Median (Q1-Q3)	26 (24-29)	26 (24-29)	26 24-29)

Table S5. Full model association between PFOS (ng/mL) and TC (mg/dL) from GAM models: adjusted β coefficients and Confidence Interval (95% CI).

**TC**				
**Parametric coefficients**	***Adolescents***		***Children***	
	**Estimate**	**95% CI**	**Estimate**	**95% CI**
(Intercept)	149.43	[146.31,152.56]	152.34	[147.38,157.3]
log(PFOS)	3.32	[2.20,4.45]	6.22	[4.32,8.13]
Citizenship: HMPC	-2.39	[-5.27,0.49]	3.55	[-3.99,11.09]
Laboratory: Legnago	2.95	[0.85,5.04]	11.15	[8.42,13.88]
Laboratory: San Bonifacio	-10.60	[-13.42,-7.78]	-9.67	[-12.34,-7]
Age class: 2	2.13	[-0.05,4.31]	3.76	[0.07,7.46]
Age class: 3	2.24	[-0.12,4.6]	4.87	[0.63,9.1]
Age class: 4	6.06	[3.43,8.7]	-0.46	[-5.8,4.88]
Age class: 5	9.11	[6.36,11.86]		
Age class: 6	10.16	[7.27,13.06]		
Sex: Males	-10.78	[-12.13,-9.43]	-0.13	[-2.21,1.95]
Physical activity: Moderate	0.35	[-2.07,2.78]	0.92	[-2.37,4.21]
Physical activity: Heavy	-3.23	[-4.8,-1.67]	1.00	[-1.38,3.38]
Smoke: Yes	-2.23	[-3.93,-0.54]		
Smoke: Ex-smoker	0.93	[-4.31,6.16]		
fruit_veg2	1.52	[0.01,3.03]	-0.35	[-2.7,2]
fruit_veg3	1.56	[-0.02,3.13]	-0.33	[-3.01,2.36]
milk_yog2	-0.36	[-1.72,1.01]	0.12	[-2.09,2.32]

milk_yog3	1.69	[-0.43,3.81]	-0.67	[-3.88,2.54]
cheese2	0.22	[-1.52,1.96]	-1.013	[-3.63,1.61]
cheese3	0.61	[-0.86,2.07]	-0.3226	[-2.83,2.19]
meat2	-0.01	[-1.36,1.33]	2.007	[-0.45,4.46]
meat3	-1.29	[-3.33,0.74]	1.2834	[-1.18,3.74]
sweet_snacks2	-1.11	[-2.62,0.4]	-1.2764	[-3.52,0.96]
sweet_snacks3	0.27	[-1.81,2.35]	0.2942	[-2.8,3.39]
eggs2				
eggs3	0.24	[-1.14,1.61]	0.1602	[-2.09,2.41]
fish2	2.26	[0.77,3.75]		
fish3	1.34	[-0.76,3.45]	-0.3187	[-2.53,1.89]
bread_pasta_cereals2			1.1481	[-1.16,3.45]
bread_pasta_cereals3	-2.29	[-4.37,-0.22]	-0.5025	[-4.1,3.09]
Salt consumption: Medium	0.48	[-0.93,1.9]	-1.7134	[-3.85,0.42]
Salt consumption: High	2.06	[-0.55,4.67]	-0.746	[-7.81,6.31]

Notes:

1. The number of age groups is different for adolescents (from 14 to 19 years old, meaning 6 age classes) and children (from 8 to 11 years old, meaning 4 age classes);
2. Children's analyses were not adjusted for smoking habits;
3. The number of quantiles for dietary habits may vary according their distribution in each subgroup.

Table S6. Full model association between PFOS (ng/mL) and SBP (mmHg) from GAM models: adjusted β coefficients and Confidence Interval (95% CI).

**SBP**				
**Parametric coefficients**	*Adolescents*		*Children*	
	**Estimate**	**95% CI**	**Estimate**	**95% CI**
(Intercept)	110.51	[108.79,112.24]	96.66	[94.47,98.86]
log(PFOS)	-0.47	[-1.02,0.08]	-0.42	[-1.18,0.33]
Citizenship: HMPC	-1.30	[-2.71,0.11]	0.38	[-2.59,3.34]
Center: Legnago	-1.68	[-2.94,-0.42]	4.49	[3.23,5.75]
Center: San Bonifacio	0.44	[-1.06,1.94]	2.78	[1.53,4.03]
Center: Noventa Vicentina	-0.21	[-1.3,0.87]	0.79	[-0.55,2.13]

Age class: 2	0.53	[-0.54,1.61]	0.31	[-1.31,1.92]
Age class: 3	1.62	[0.46,2.78]	2.14	[0.34,3.95]
Age class: 4	2.92	[1.62,4.22]	2.01	[-0.2,4.22]
Age class: 5	4.69	[3.33,6.06]		
Age class: 6	4.97	[3.53,6.42]		
Sex: Males	6.59	[5.93,7.26]	-0.22521	[-1.05,0.6]
Physical activity: Moderate	-0.54	[-1.73,0.65]	0.70847	[-0.59,2]
Physical activity: Heavy	-0.92	[-1.69,-0.16]	0.12938	[-0.82,1.08]
Smoke: Yes	-1.90	[-2.73,-1.07]		
Smoke: Ex-smoker	0.02	[-2.55,2.6]		
fruit_veg2	0.39	[-0.35,1.13]	0.40	[-0.53,1.32]
fruit_veg3	-0.04	[-0.81,0.73]	-0.19	[-1.25,0.87]
milk_yog2	0.02	[-0.65,0.68]	-0.06141	[-0.93,0.81]
milk_yog3	-0.43	[-1.47,0.6]	-0.06	[-1.32,1.21]
cheese2	0.74	[-0.11,1.59]	0.49	[-0.54,1.53]
cheese3	0.84	[0.12,1.55]	0.38	[-0.61,1.36]
meat2	1.14	[0.48,1.79]	-0.37	[-1.34,0.59]
meat3	2.23	[1.23,3.22]	0.02	[-0.95,0.99]
sweet_snacks2	-0.40	[-1.14,0.34]	-0.06285	[-0.95,0.82]
sweet_snacks3	0.29	[-0.73,1.31]	-0.46635	[-1.69,0.76]
eggs2				
eggs3	0.01	[-0.66,0.69]	0.06357	[-0.83,0.95]
fish2	0.29	[-0.44,1.02]		
fish3	0.12	[-0.91,1.15]	-0.11243	[-0.98,0.76]
bread_pasta_cereals2			0.53261	[-0.4,1.46]
bread_pasta_cereals3	-2.19	[-3.21,-1.17]	-1.97719	[-3.41,-0.54]
Salt consumption: Medium	-0.74	[-1.44,-0.05]	0.72854	[-0.11,1.57]
Salt consumption: High	-1.17	[-2.44,0.11]	0.58486	[-2.19,3.36]

Table S7. Full model association between PFOS (ng/mL) and BMI z-score from GAM models: adjusted β coefficients and Confidence Interval (95% CI).

**BMI z-score**				
**Parametric coefficients**	***Adolescents***		***Children***	
	**Estimate**	**95% CI**	**Estimate**	**95% CI**
(Intercept)	0.67	[0.54,0.81]	0.84	[0.58,1.1]
log(PFOS)	-0.11	[-0.16,-0.06]	-0.27	[-0.36,-0.17]
Citizenship: HMPC	-0.01	[-0.13,0.12]	-0.21	[-0.58,0.17]
Center: Legnago	-0.08	[-0.16,0]	0.14	[-0.01,0.29]
Center: San Bonifacio	0.06	[-0.03,0.15]	0.27	[0.12,0.41]
Center: Noventa Vicentina	0.05	[-0.03,0.13]	0.33	[0.18,0.48]
Age class: 2	-0.14	[-0.23,-0.05]	-0.28	[-0.46,-0.09]
Age class: 3	-0.23	[-0.33,-0.13]	-0.29	[-0.5,-0.08]
Age class: 4	-0.35	[-0.46,-0.24]	-0.29	[-0.55,-0.02]
Age class: 5	-0.42	[-0.53,-0.31]		
Age class: 6	-0.53	[-0.65,-0.42]		
Sex: Males	0.15	[0.09,0.21]	0.03	[-0.08,0.13]
Physical activity: Moderate	0.02	[-0.08,0.13]	0.06	[-0.11,0.22]
Physical activity: Heavy	-0.02	[-0.09,0.05]	-0.14	[-0.26,-0.02]
Smoke: Yes	0.12	[0.05,0.2]		
Smoke: Ex-smoker	0.11	[-0.12,0.34]		
fruit_veg2	0.06	[-0.01,0.13]	0.13	[0.01,0.24]
fruit_veg3	0.14	[0.07,0.21]	0.17	[0.04,0.3]
milk_yog2	0.00	[-0.06,0.06]	0.08	[-0.03,0.18]
milk_yog3	-0.18	[-0.27,-0.09]	0.04	[-0.12,0.2]
cheese2	0.00	[-0.08,0.08]	0.08	[-0.05,0.21]
cheese3	-0.02	[-0.09,0.04]	-0.01	[-0.13,0.11]
meat2	0.04	[-0.02,0.1]	0.07	[-0.05,0.19]
meat3	0.05	[-0.04,0.14]	0.19	[0.07,0.31]
sweet_snacks2	-0.19	[-0.25,-0.12]	-0.04	[-0.15,0.07]
sweet_snacks3	-0.22	[-0.32,-0.13]	-0.12	[-0.28,0.03]

eggs2				
eggs3	-0.07	[-0.13,-0.01]	0.03	[-0.08,0.14]
fish2	0.03	[-0.04,0.1]		
fish3	0.04	[-0.05,0.13]	0.12	[0.01,0.23]
bread_pasta_cereals2			-0.05	[-0.16,0.07]
bread_pasta_cereals3	-0.03	[-0.12,0.06]	-0.09	[-0.27,0.09]
Salt consumption: Medium	0.00	[-0.07,0.06]	-0.05	[-0.16,0.05]
Salt consumption: High	-0.07	[-0.19,0.04]	-0.07	[-0.42,0.28]

Table S8. Association between PFAS (ng/mL) and serum lipids (mg/dL) from GAM models, stratified by gender: adjusted β* coefficients and 95% Confidence Intervals (CIs). ADOLESCENTS

PFAS	TC						HDL						LDL						Tryglicerides					
	M			F			M			F			M			F			M			F		
	Coef	CI Lower	CI Upper	Coef	CI Lower	CI Upper	Coef	CI Lower	CI Upper	Coef	CI Lower	CI Upper	Coef	CI Lower	CI Upper	Coef	CI Lower	CI Upper	Coef	CI Lower	CI Upper	Coef	CI Lower	CI Upper
log_pfoa	1.03	-0.01	2.07	1.01	-0.06	2.08	-0.265	-0.670	0.139	-0.04	-0.50	0.42	1.206	0.296	2.116	0.74	-0.15	1.63	0.008	-0.012	0.027	0.02	0.00	0.04
II Q	-1.42	-3.99	1.16	3.13	0.67	5.60	-0.044	-1.048	0.961	-0.10	-1.15	0.96	-1.026	-3.287	1.236	2.67	0.61	4.72	-0.024	-0.072	0.024	0.05	0.01	0.09
III Q	0.24	-2.40	2.87	2.64	-0.01	5.28	-0.010	-1.039	1.019	0.03	-1.10	1.17	0.391	-1.923	2.705	2.08	-0.13	4.29	-0.019	-0.068	0.030	0.03	-0.01	0.07
IV Q	2.98	0.26	5.69	2.49	-0.50	5.49	-0.390	-1.450	0.669	-0.04	-1.33	1.24	2.990	0.605	5.375	2.08	-0.42	4.57	0.025	-0.026	0.075	0.04	-0.01	0.08
log_pfos	3.63	2.10	5.16	3.19	1.55	4.84	1.003	0.406	1.599	1.47	0.77	2.18	3.187	1.863	4.510	2.13	0.76	3.51	-0.025	-0.053	0.004	-0.02	-0.04	0.01
II Q	0.99	-1.57	3.55	6.21	3.75	8.67	0.639	-0.363	1.640	1.12	0.07	2.18	1.106	-1.145	3.356	4.70	2.65	6.75	-0.040	-0.088	0.007	0.03	-0.01	0.07
III Q	2.92	0.30	5.53	5.06	2.45	7.67	1.173	0.151	2.195	1.26	0.14	2.38	2.668	0.374	4.963	3.42	1.24	5.59	-0.053	-0.102	-0.004	0.01	-0.03	0.05
IV Q	6.10	3.43	8.78	4.99	2.10	7.88	1.558	0.512	2.604	2.30	1.06	3.54	5.129	2.795	7.464	3.58	1.17	5.99	-0.031	-0.081	0.019	-0.05	-0.09	-0.01
log_pfhxs	1.43	0.22	2.64	1.43	0.14	2.72	-0.093	-0.566	0.380	0.06	-0.49	0.61	1.535	0.471	2.598	1.15	0.08	2.23	0.000	-0.022	0.023	0.01	0.00	0.03
II Q	-0.01	-2.64	2.61	3.21	0.81	5.62	0.145	-0.879	1.169	-0.54	-1.57	0.49	0.078	-2.229	2.385	3.28	1.28	5.29	-0.024	-0.073	0.025	0.04	0.00	0.07

III Q	0.92	-1.69	3.53	2.09	-0.46	4.64	0.195	-0.823	1.213	0.00	-1.09	1.10	0.908	-1.379	3.196	1.66	-0.47	3.79	-0.032	-0.080	0.017	0.03	-0.01	0.06
IV Q	3.48	0.80	6.16	3.18	0.20	6.16	0.153	-0.892	1.198	0.28	-0.99	1.56	3.393	1.040	5.746	2.97	0.49	5.46	-0.004	-0.054	0.046	0.01	-0.04	0.05
PFNA low	4.84	2.82	6.86	2.01	-0.26	4.28	0.744	-0.046	1.534	1.56	0.59	2.53	4.325	2.551	6.098	0.26	-1.64	2.15	-0.010	-0.048	0.028	0.02	-0.02	0.05
PFNA medium	4.91	2.55	7.26	2.49	-0.12	5.09	1.153	0.231	2.076	1.97	0.85	3.08	4.499	2.441	6.556	1.62	-0.55	3.78	-0.023	-0.067	0.021	-0.05	-0.09	-0.01

Table S9. Association between PFAS (ng/mL) and serum lipids (mg/dL) from GAM models, stratified by gender: adjusted β* coefficients and 95% Confidence Intervals (CIs). CHILDREN

PFAS	TC						HDL						LDL						Tryglicerides					
	M			F			M			F			M			F			M			F		
	Coef	CI Lower	CI Upper	Coef	CI Lower	CI Upper	Coef	CI Lower	CI Upper	Coef	CI Lower	CI Upper	Coef	CI Lower	CI Upper	Coef	CI Lower	CI Upper	Coef	CI Lower	CI Upper	Coef	CI Lower	CI Upper
log_pfoa	0.33	-1.49	2.15	1.35	-0.50	3.20	0.95	0.15	1.75	0.33	-0.42	1.09	-0.39	-1.99	1.22	0.80	-0.87	2.47	-0.02	-0.05	0.01	0.01	-0.01	0.04
II Q	-4.63	-8.87	-0.39	4.74	0.76	8.71	0.90	-0.96	2.75	0.39	-1.24	2.02	-4.98	-8.71	-1.25	3.60	0.02	7.19	-0.06	-0.12	0.01	0.04	-0.01	0.10
III Q	-2.13	-6.32	2.07	2.54	-1.51	6.60	1.88	0.05	3.71	0.76	-0.90	2.43	-3.57	-7.25	0.12	1.08	-2.58	4.75	-0.05	-0.11	0.02	0.03	-0.03	0.09
IV Q	0.27	-3.84	4.39	5.45	1.13	9.77	1.75	-0.05	3.56	1.07	-0.70	2.85	-0.65	-4.28	2.97	3.81	-0.09	7.71	-0.07	-0.13	0.00	0.04	-0.02	0.11
log_pfos	4.50	1.74	7.25	8.01	5.34	10.67	2.01	0.80	3.22	1.88	0.78	2.98	3.26	0.82	5.70	5.75	3.34	8.17	-0.04	-0.08	0.01	0.01	-0.03	0.05
II Q	2.10	-1.89	6.08	5.32	1.46	9.18	2.90	1.16	4.63	1.74	0.15	3.33	0.10	-3.41	3.61	3.59	0.09	7.09	-0.04	-0.10	0.02	-0.01	-0.06	0.05
III Q	3.29	-0.85	7.43	7.98	3.97	11.99	2.36	0.54	4.18	2.56	0.91	4.21	1.50	-2.17	5.16	4.86	1.22	8.49	-0.01	-0.08	0.05	0.02	-0.03	0.08
IV Q	6.71	2.66	10.76	10.08	6.06	14.10	3.03	1.25	4.82	2.98	1.32	4.63	5.03	1.43	8.62	6.81	3.18	10.45	-0.07	-0.13	-0.01	-0.01	-0.06	0.05
log_pfhxs	-0.13	-2.39	2.13	2.68	0.44	4.92	1.06	0.06	2.05	0.50	-0.42	1.42	-0.83	-2.83	1.17	2.02	0.00	4.04	-0.03	-0.07	0.00	0.01	-0.02	0.04
II Q	-4.37	-8.49	-0.24	1.48	-2.39	5.34	1.48	-0.32	3.28	-0.61	-2.19	0.97	-5.00	-8.63	-1.38	0.99	-2.50	4.49	-0.06	-0.12	0.00	0.06	0.01	0.12

III Q	-2.68	-6.94	1.57	3.12	-0.91	7.15	2.19	0.32	4.05	1.17	-0.48	2.82	-4.69	-8.43	-0.94	1.81	-1.83	5.45	-0.01	-0.08	0.05	0.01	-0.04	0.07
IV Q	-1.08	-5.27	3.12	4.75	0.54	8.96	2.04	0.20	3.88	0.56	-1.16	2.28	-2.03	-5.72	1.67	3.60	-0.21	7.40	-0.08	-0.15	-0.02	0.04	-0.02	0.10
PFNA low	2.73	-0.80	6.25	3.05	-0.70	6.81	0.95	-0.60	2.50	1.91	0.37	3.45	2.34	-0.77	5.45	0.50	-2.90	3.89	-0.03	-0.09	0.02	0.02	-0.04	0.07
PFNA medium	6.07	0.56	11.59	8.72	3.64	13.81	1.42	-1.00	3.83	2.10	0.02	4.18	5.14	0.28	10.00	6.79	2.20	11.38	-0.02	-0.10	0.06	-0.02	-0.09	0.06

Table S10. Association between PFAS (ng/mL) and blood pressure (mmHg) from GAM models, stratified by gender: adjusted β* coefficients and 95% Confidence Intervals (CIs). ADOLESCENTS

	SYSTOLIC BP						DIASTOLIC BP					
**PFAS**	**M**			**F**			**M**			**F**		
	**Coef**	**CI Lower**	**CI Upper**	**Coef**	**CI Lower**	**CI Upper**	**Coef**	**CI Lower**	**CI Upper**	**Coef**	**CI Lower**	**CI Upper**
ln_pfoa	-0.04	-0.59	0.50	-0.39	-0.88	0.10	-0.06	-0.44	0.32	-0.18	-0.53	0.17
II Q	-0.16	-1.51	1.18	-0.52	-1.66	0.61	-0.05	-1.00	0.90	-0.48	-1.29	0.32
III Q	-0.60	-1.98	0.78	-1.34	-2.55	-0.12	-0.33	-1.30	0.64	-0.22	-1.08	0.64
IV Q	-0.21	-1.63	1.21	-0.88	-2.26	0.49	0.17	-0.83	1.17	-0.46	-1.43	0.52
log_pfos	-0.21	-1.02	0.59	-0.93	-1.69	-0.18	-0.25	-0.81	0.32	-0.59	-1.12	-0.05
II Q	0.08	-1.26	1.42	-1.05	-2.18	0.08	0.18	-0.77	1.12	-1.10	-1.90	-0.30
III Q	-0.70	-2.07	0.67	-1.17	-2.37	0.02	-0.31	-1.27	0.66	-1.03	-1.88	-0.18
IV Q	-0.60	-2.00	0.80	-2.36	-3.69	-1.03	-0.54	-1.53	0.44	-0.82	-1.76	0.12
log_pfhxs	-0.27	-0.91	0.36	-0.33	-0.92	0.26	-0.14	-0.59	0.30	-0.20	-0.62	0.22
II Q	-0.34	-1.71	1.03	-0.26	-1.37	0.84	-0.03	-1.00	0.93	-0.04	-0.82	0.74
III Q	-0.75	-2.11	0.62	-0.69	-1.86	0.48	-0.24	-1.20	0.72	-0.15	-0.98	0.68
IV Q	-0.56	-1.95	0.84	-0.43	-1.80	0.93	-0.47	-1.46	0.51	-0.08	-1.05	0.89
PFNA low	-0.31	-1.37	0.75	-1.03	-2.07	0.02	0.26	-0.48	1.01	0.52	-0.22	1.26
PFNA medium	-0.03	-1.27	1.20	-0.85	-2.05	0.34	0.38	-0.49	1.25	-0.23	-1.07	0.62

Table S11. Association between PFAS (ng/mL) and blood pressure (mmHg) from GAM models, stratified by gender: adjusted β* coefficients and 95% Confidence Intervals (CIs). CHILDREN

	SYSTOLIC BP						DIASTOLIC BP					
PFAS	M			F			M			F		
	Coef	CI Lower	CI Upper	Coef	CI Lower	CI Upper	Coef	CI Lower	CI Upper	Coef	CI Lower	CI Upper
ln_pfoa	-0.28	-0.97	0.41	-0.90	-1.64	-0.17	0.43	-0.11	0.97	-0.23	-0.79	0.33
II Q	0.02	-1.60	1.63	-0.44	-2.03	1.15	0.78	-0.49	2.04	0.09	-1.11	1.29
III Q	0.42	-1.18	2.01	-1.11	-2.73	0.51	0.33	-0.92	1.58	0.13	-1.10	1.35
IV Q	-0.69	-2.26	0.88	-1.62	-3.34	0.11	1.17	-0.06	2.39	-0.15	-1.46	1.15
log_pfos	-0.49	-1.54	0.56	-0.53	-1.61	0.55	0.24	-0.58	1.07	-0.19	-1.01	0.62
II Q	-0.74	-2.26	0.78	0.11	-1.45	1.67	1.01	-0.18	2.20	0.29	-0.88	1.46
III Q	-0.16	-1.75	1.42	0.12	-1.50	1.73	1.52	0.29	2.76	0.14	-1.07	1.36
IV Q	-0.75	-2.30	0.80	-1.15	-2.77	0.48	0.51	-0.70	1.72	-0.74	-1.96	0.48
log_pfhxs	-0.43	-1.29	0.44	-1.18	-2.08	-0.29	0.52	-0.15	1.19	-0.33	-1.01	0.34
II Q	0.32	-1.26	1.90	-1.48	-3.02	0.06	0.30	-0.93	1.54	0.96	-0.21	2.12
III Q	-0.38	-2.01	1.25	-2.66	-4.27	-1.05	0.63	-0.64	1.90	-0.57	-1.79	0.65
IV Q	-0.60	-2.20	1.01	-1.86	-3.54	-0.18	0.73	-0.52	1.99	-0.32	-1.59	0.96
PFNA low	-0.70	-2.05	0.64	-0.12	-1.62	1.39	0.80	-0.25	1.84	-0.29	-1.42	0.85
PFNA medium	0.75	-1.35	2.85	-1.23	-3.27	0.80	-0.26	-1.90	1.38	-1.60	-3.13	-0.07

Figure S1. Directed acyclic graph (DAG) for the selection of covariates.

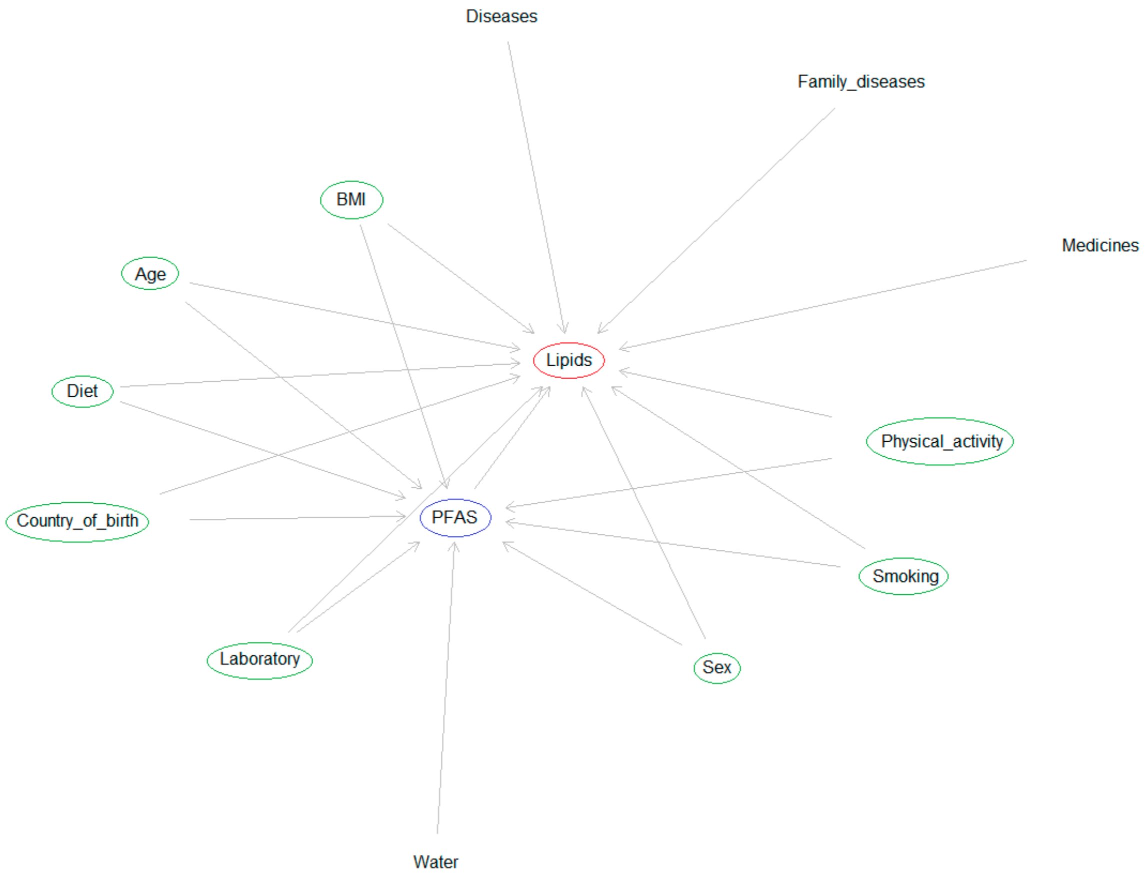

Figure S2. Flow-chart of study population.

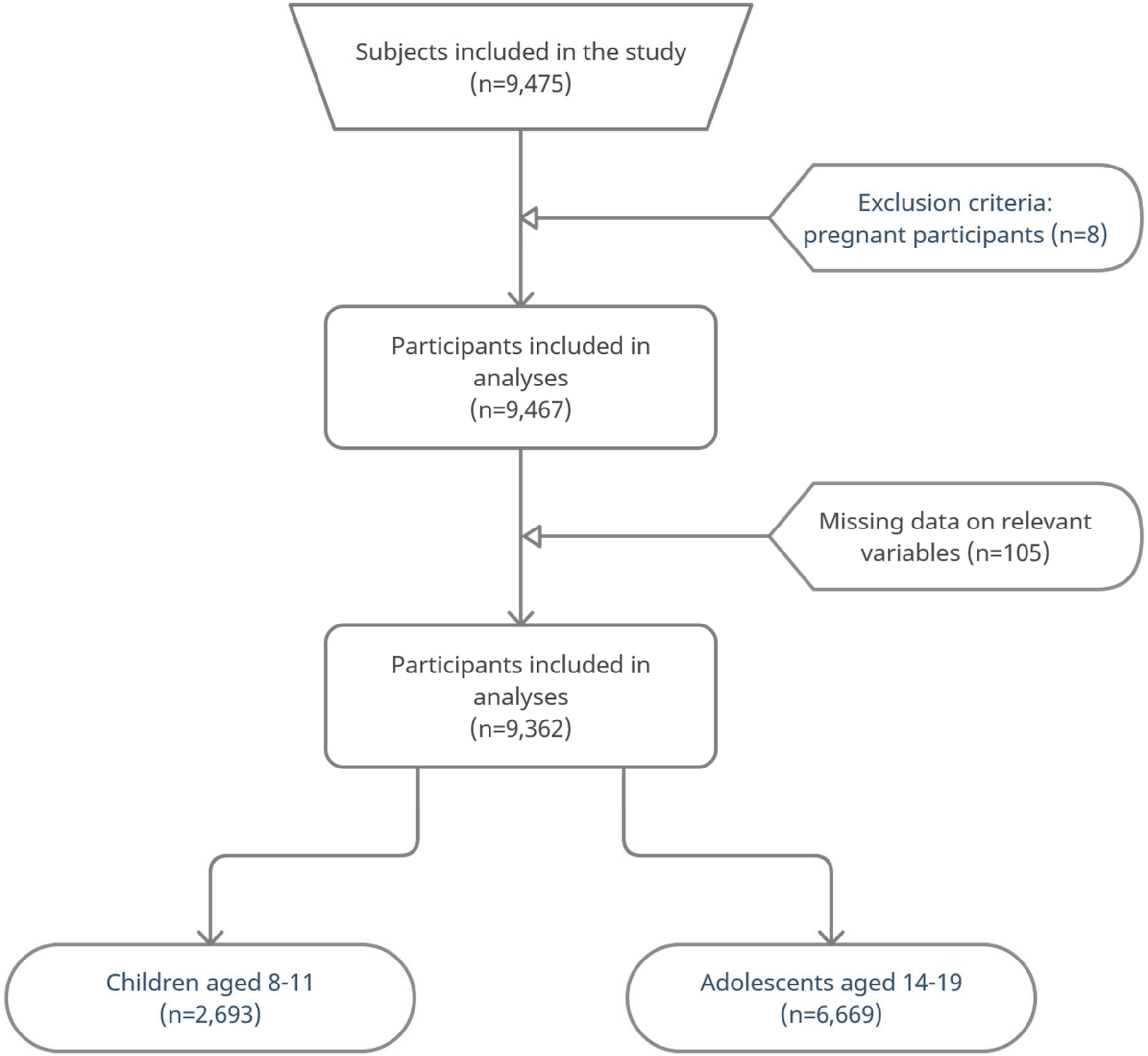

Supplement: Supplementary file 1 [file ijerph-18-12881-s001.zip › ijerph-1451745-supplementary.pdf]
